# Efficacy of Platelet-Rich Plasma and Platelet-Rich Fibrin in Enhancing Dental Implant Osseointegration

**DOI:** 10.31661/gmj.v13iSP1.3679

**Published:** 2024-12-08

**Authors:** Emad Taghizadeh, Sahar Negargar, Sara Noorizadeh, Seyed Mohammad Mahdi Mirmohammadi, Zahra Salmani

**Affiliations:** ^1^ Department of Oral and Maxillofacial Surgery, Faculty of Dentistry, Shahed University, Tehran, Iran; ^2^ Department of Dentistry Asad Abadi Hospital, Tabriz, Iran; ^3^ Department of Periodontics, Faculty of Dentistry, Shahed University, Tehran, Iran; ^4^ Department of Oral and Maxillofacial Surgery, Shahid Beheshti University of Medical Sciences, Tehran, Iran; ^5^ Department of Periodontics, Faculty of Dentistry, Alborz University of Medical Sciences, Karaj, Iran

**Keywords:** Dental Implants, Osseointegration, Platelet-Rich Plasma (PRP), Platelet-Rich Fibrin (PRF), Bone Regeneration

## Abstract

The successful integration of dental implants relies on osseointegration, a process essential for implant stability and longevity. Platelet-Rich Plasma (PRP) and Platelet-Rich Fibrin (PRF) have gained attention as biological enhancers for this process due to their high concentrations of growth factors that promote bone regeneration and accelerate healing. This review assesses the efficacy of PRP and PRF in enhancing osseointegration by exploring their biological mechanisms, clinical applications, and advantages for patients with compromised bone or healing potential. Literature indicates that PRP and PRF can improve initial implant stability and accelerate healing. PRP’s platelet-derived growth factors (e.g., PDGF, TGF-β, VEGF) stimulate cellular proliferation and angiogenesis, critical for early bone healing. PRF’s fibrin-rich matrix provides a sustained release of these factors, supporting prolonged tissue regeneration and soft tissue repair. However, challenges remain, including variability in preparation methods and limited long-term data, underscoring the need for standardized protocols and further research. In conclusion, PRP and PRF demonstrate promise as adjuncts for enhancing dental implant osseointegration, particularly in complex cases. With more evidence and established protocols, they have the potential to become standard tools in implant dentistry, offering improved outcomes and greater predictability in patient care.

## Introduction

Dental implants have become an essential treatment modality for patients with missing teeth, offering durable and functional alternatives that restore oral aesthetics and function [1].

The success of dental implants fundamentally depends on osseointegration, the biological process linking bone to the implant’s surface, which is crucial for implant stability and longevity [2]. Achieving reliable osseointegration can be challenging, especially in patients with compromised bone quality or health conditions that may impede bone healing. [3][4].

This process is essential for the long-term stability of implants and ultimately impacts their success and longevity [5]. Osseointegration involves complex interactions between the implant material, bone cells, and the surrounding biological environment [6]. For optimal outcomes, this integration requires sufficient bone-to-implant contact and rapid bone healing, which can be challenging in cases with compromised bone quality or limited healing potential [7].

Platelet-rich plasma (PRP) and platelet-rich Fibrin (PRF) have emerged as promising biological adjuncts in dental implantology due to their potential to accelerate and enhance osseointegration [8]. Both of them are autologous platelet concentrate and growth factors but differ in preparation method and composition. [9]. These factors play essential roles in promoting cellular migration, proliferation, and differentiation, all of which are critical in bone healing and tissue regeneration [10]. PRP has been widely explored in various medical fields, including orthopedics, sports medicine, and maxillofacial surgery, where it is used to stimulate healing and reduce recovery time [11]. On the other hand, PRF has gained popularity in dental applications, particularly for enhancing bone regeneration around implants and accelerating soft tissue healing due to its simple preparation method, low cost, and efficacy in promoting tissue repair [12].

This review aims to critically evaluate the efficacy of PRP and PRF in enhancing dental implant osseointegration, examining existing clinical on their biological effects and clinical outcomes. It will explore the underlying mechanisms of both in bone regeneration, compare their relative advantages, and discuss factors that may influence their efficacy in clinical applications.

## Biological Mechanisms of PRP and PRF

PRP and PRF are believed to enhance osseointegration by accelerating the initial phases of bone healing through the delivery of concentrated growth factors and cytokines directly at the implant site [13]. This augmentation of biological activity fosters an environment conducive to cell recruitment, proliferation, and differentiation, which are critical to the formation of new bone around implants [14]. These biomaterials function as both a scaffold for cellular activity and as a reservoir for bioactive molecules that activate signaling pathways instrumental to tissue repair and regeneration [15].

PRP, derived from autologous blood processed to concentrate platelets, is rich in growth factors that are rapidly released upon activation. Key growth factors present in PRP include Platelet-Derived Growth Factor (PDGF), which promotes cell proliferation and chemotaxis, and Transforming Growth Factor-Beta (TGF-β), which stimulates the differentiation of mesenchymal stem cells (MSCs) into osteoblasts, the primary cells involved in bone formation [16]. Vascular Endothelial Growth Factor (VEGF) within PRP contributes to angiogenesis, the formation of new blood vessels, which is essential for providing oxygen and nutrients to regenerating tissue and supporting early wound healing around implants [17]. Together, these factors support a cascade of cellular events that enhance the initial stages of osseointegration, contributing to a faster and more stable bone-to-implant interface [18].

PRF, while similar in composition to PRP, offers unique biological advantages due to its preparation method, which results in a fibrin-rich matrix that entraps platelets, leukocytes, and cytokines [19]. This fibrin network, formed without the use of anticoagulants, provides a scaffold that not only promotes cellular migration and attachment but also supports a sustained release of growth factors over an extended period [20].

This gradual release mechanism, combined with the fibrin matrix, enhances PRF’s regenerative potential. Growth factors in PRF, such as Insulin-Like Growth Factor (IGF), Epidermal Growth Factor (EGF), and TGF-β, are crucial for osteoblastic differentiation, while PDGF and VEGF play roles in cellular migration and angiogenesis [13]. Additionally, the presence of leukocytes in PRF supports an anti-inflammatory response that mitigates infection risk, and a potential complication in implant placement, and promotes a more favorable healing environment [14].

Cellular mechanisms activated by PRP and PRF extend beyond initial cell recruitment and growth factor signaling. For example, these platelet concentrates activate osteoblasts and osteoclasts, the cells responsible for bone formation and remodeling, respectively, creating a dynamic balance that is essential for bone regeneration [21]. MSCs attracted to the implant site differentiate into osteoblasts under the influence of TGF-β and PDGF, which results in increased bone formation and mineralization around the implant [22]. Furthermore, PRP and PRF have been shown to enhance the expression of osteogenic markers, such as alkaline phosphatase and osteocalcin, that are critical in the maturation of new bone tissue [20]. This cellular activity is critical for establishing a strong and durable connection between the bone and implant surface, ultimately improving the stability and success rates of dental implants [18].

## Clinical Applications in Dental Implantology

Bone Regeneration and Implant Stability

Evidence suggests PRF can enhance bone density and implant stability, especially in high-aesthetic demand zones.[23] For example, a study by Deb et al. [1] found that PRF-treated sites showed greater bone density and stability than controls, highlighting PRF’s potential to accelerate healing in challenging areas.

Also, injectable PRF (i-PRF) demonstrated positive effects on bone formation and soft tissue healing by sustaining growth factor release, proving particularly beneficial in cases where gradual, long-term regeneration is needed [24]. In a trial focusing on maxillary anterior implants, Boora et al. [25] found that PRF treatment reduced marginal bone loss within three months, highlighting PRF’s role in improving early implant stability.

Another trial involving PRP in combination with demineralized freeze-dried bone allograft (DFDBA) noted enhanced clinical attachment levels and reduced probing depth in periodontal defects. However, PRP’s efficacy did not significantly exceed that of DFDBA alone, indicating that PRP’s advantages may vary depending on the type of graft material used [26].

Soft Tissue Healing and Prolonged Regenerative Support

PRF derivatives, such as advanced PRF (A-PRF), have shown additional value in applications requiring long-term healing support.[3] For example, a multi-arm randomized trial found that A-PRF combined with freeze-dried bone allograft significantly reduced ridge height loss following tooth extraction, underscoring its potential for maintaining alveolar bone dimensions over time [27]. Also, Zeitounlouian et al. [28] found that while i-PRF effectively supported alveolar bone preservation during orthodontic tooth movement, it showed no significant difference in bone preservation compared to control groups, suggesting that i-PRF may be particularly suited to soft tissue repair rather than supporting bone under mechanical stress.

Applications in Low Bone Density or Compromised Quality Cases

Tabrizi et al. [29] conducted a split-mouth trial that revealed significantly higher stability in PRF-treated implants as measured by resonance frequency analysis, indicating PRF’s potential to enhance early osseointegration in low-density bone regions. A comparison between PRF and Concentrated Growth Factors (CGF) in immediate implants further supports PRF’s efficacy [30].

Gaur et al. [31] reported that both PRF and CGF offered similar improvements in implant stability, particularly noticeable 12-16 weeks post-application, highlighting their role in promoting implant stability and supporting osseointegration.

## Practical Implications

Together, these studies suggest that PRP and PRF offer valuable adjunctive benefits in implantology, particularly for enhancing bone regeneration, implant stability, and soft tissue healing [2]. PRF, with its sustained growth factor release, appears especially useful in high-demand zones and in cases requiring prolonged regenerative support [23].

Studies suggest that PRF, with its extended growth factor release, can be particularly effective in cases with compromised bone quality, such as low-density bone regions like the posterior maxilla [32]. PRF and i-PRF may provide enhanced implant stability, reduced healing time, and improved regenerative support, making them valuable in complex cases where bone quality or healing potential is limited [33][34].

## Comparison of PRP and PRF

**Table:** Table1. Comparison of PRP and PRF

**Parameter**	**PRP (Platelet-Rich Plasma) **	**PRF (Platelet-Rich Fibrin) **	**Author(s)**
Preparation Complexity	Requires anticoagulant, multiple processing steps	Simpler, requires no anticoagulant	Giannini et al. [40]
Growth Factor Release Timing	Initial high release within the first 15-60 minutes	Sustained release over 10 days	
	**Growth Factor** **Released means (pg/ml) with ranges from 0-10 days **		
PDGF-AA	6176 (2812-9184)	9262 (2877-13839)	
PDGF-AB	4131 (1837-5492)	4396 (862-7563)	
PDGF-BB	1155 (531-1371)	680 (220-1147)	Kobayashi et al. [30]
TGF-beta1	1105 (619-1453)	1110 (302-1714)	
VEGF	847 (693-1009)	732 (537-914)	
EGF	363 (210-497)	512 (146-715)	
IGF	54 (44-67) 166	(55-252)	
Antimicrobial Efficacy	Moderate	High	Zafar et al. [41]
Bone Regeneration (Apical Closure Success)	85.1%	85.2%	Rizk et al. [32]
Dentine Bridge Formation in Pulp Capping volume (mean ± SD)	0.1392±0.0161 mm ^3^	0.1368±0.0128 mm ^3^	Shekar et al. [38]
	**Tissue Regeneration in Periodontal Applications ** **Mean ± SD After 9 months **		
PPD	4.25±0.5	4±0.34	Suchetha et al. [36]
CAL	3.35±0.85	3.25±0.91	
BL	4.2±0.44	4.15±0.41	

**PDGF:** Platelet-derived growth factor, **TGFB1:** transforming growth factor beta 1, **VEGF:** vascular endothelial growth factor, **EGF:** epidermal growth factor, and **IGF:** insulin-like growth factor. **PPD:** probing pocket depth; **CAL:** clinical attachment level; **BL:** Bone level

**Table:** Table2. Factors Influencing PRP and PRF Efficacy

**Factor**	**Influence on PRP**	**Influence on PRF**	**Clinical Implications**
**Age**	Reduced efficacy in older patients	Lower fibrin density with age	Adjust dosage and protocol for age differences
**Bone Quality & Density**	Limited regeneration in poor bone	Enhanced stability in dense bone	Choose PRF for cases with low bone density
**Systemic Health**	Lower response in chronic conditions	Consistent healing in comorbidities	Use PRF for patients with systemic diseases
**Implant Location & Vascularity**	High release in vascular areas	Sustained release in low vascularity	Select based on site vascularity
**Technical Aspects**	Requires anticoagulant and handling	Simple preparation without additives	Fewer errors and ease of use with PRF

**Figure-1:**
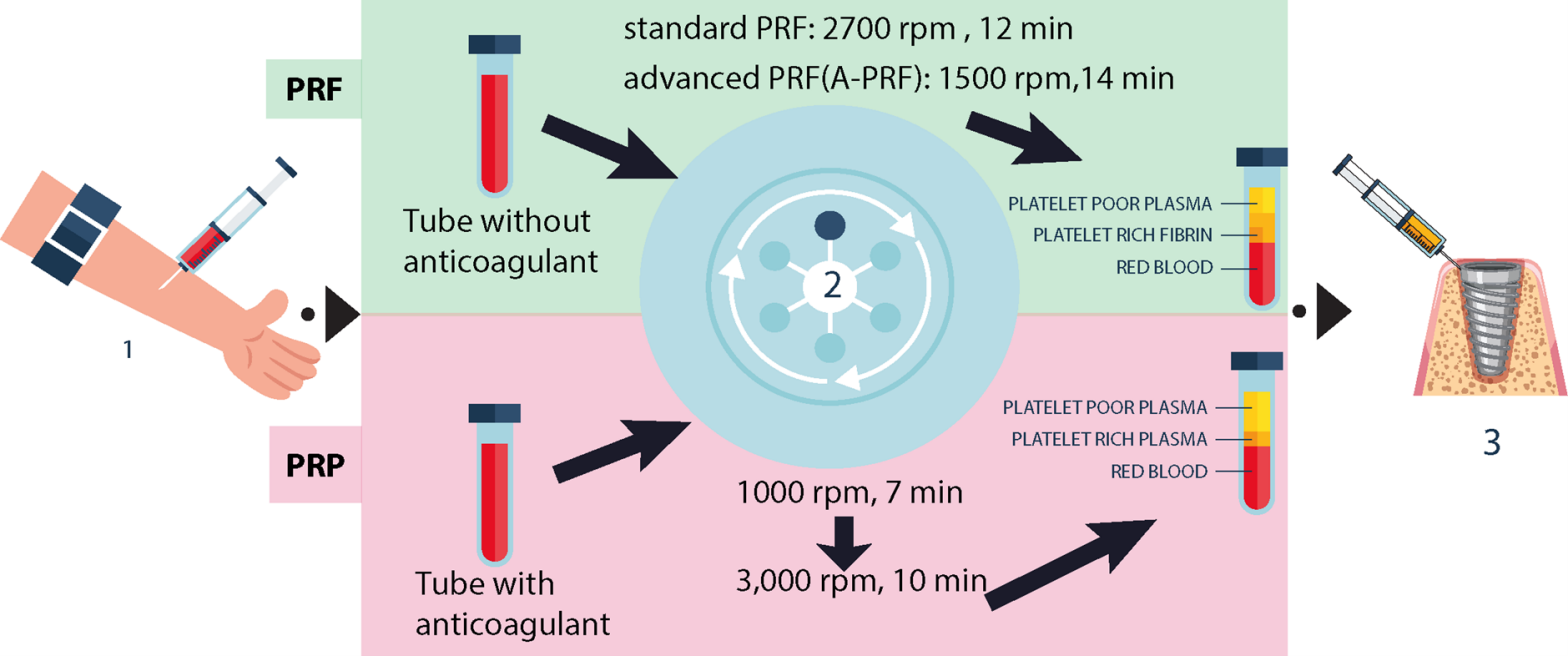
Figure 1. Schematic Representation of PRP and PRF Preparation Protocols; 1: Blood Collection: Blood (10 cc) is drawn from the patient’s arm and placed into separate tubes for PRP or PRF preparation. 2: Centrifugation Process: PRF: Blood is placed in a tube without an anticoagulant and centrifuged at either 2700 rpm for 12 minutes (standard PRF) or 1500 rpm for 14 minutes (A-PRF). PRP: Blood is placed in a tube with an anticoagulant and subjected to a two-step centrifugation process; first at 1000 rpm for 7 minutes, followed by 3000 rpm for 10 minutes. 3: Application: The PRP or PRF concentrate is applied to the implant site.

Figure-1 illustrates a schematic of the PRP and PRF protocols. PRP and PRF protocols differ in centrifugation steps, anticoagulant use, and growth factor release timing [30]. PRP requires two centrifugation steps and an anticoagulant to prevent clotting, yielding a rapid release of growth factors beneficial for acute healing needs [35]. PRF, however, involves a single, lower-speed centrifugation without anticoagulants, resulting in a natural fibrin clot that gradually releases growth factors over time, supporting sustained tissue regeneration [30].

PRP releases higher concentrations of growth factors within the first 60 minutes post-application, making it suitable for treatments requiring immediate results [30]. Conversely, PRF, due to its fibrin matrix, provides a sustained release of growth factors over several days, which supports prolonged healing needs [30]. This continuous release profile is also evident in periodontal regeneration studies, where PRF is noted to aid in long-term tissue regeneration without the need for repeated applications [36].

Table-1 provides a comparison of PRP and PRF across several applications and performance parameters based on empirical studies.

Studies that compare both of them have demonstrated similar overall efficacy, yet with notable distinctions in specific applications.[32] For example, a study on canine retraction in orthodontics reported that PRP accelerated canine movement more rapidly than PRF initially, although long-term differences were minimal [37]. Another study on direct pulp capping in teeth exposed to caries found both are effective, with no significant difference in their ability to stimulate dentine bridge formation [38].

In the context of bone regeneration, PRF has shown advantages in providing stable support and scaffold structure due to its dense fibrin matrix. This matrix facilitates new bone growth more effectively in some settings than PRP [20]. As an example, a study evaluating socket preservation after tooth extraction showed that both methods combined with a collagen plug effectively preserved socket dimensions, with PRF proving beneficial for maintaining bone density and height over the long term [39]. Similarly, another study on immature permanent teeth demonstrated that PRF was slightly more effective than PRP in achieving apical closure and root lengthening over 12 months [32].

PRP requires an anticoagulant and two centrifugation steps, then releases growth factors almost immediately upon activation, which is beneficial for acute regenerative needs [30].

On the other hand, PRF does not require additives, simplifying preparation and reducing the risk of contamination. [30] So, PRF preparation is less prone to error, with fewer manipulation steps, which can make it advantageous in settings where ease of preparation and low variability are critical [40].

Furthermore, PRF has demonstrated superior antimicrobial efficacy compared to PRP in studies that added nano-silver particles to both compounds, possibly due to PRF’s denser fibrin network, which can hold antibacterial agents more effectively [41]. This quality may benefit wound healing by minimizing infection risks, especially in periodontal and implant-related surgeries.

Factors Influencing the Efficacy of PRP and PRF

The effectiveness of PRP and PRF in enhancing dental implant osseointegration is influenced by a variety of biological, clinical, and technical factors [23][42]. Understanding these variables is essential for optimizing outcomes and maximizing the regenerative potential of these autologous biomaterials [1]. Key influencing factors include patient-specific variables such as age, bone quality, implant location, and systemic health, alongside technical considerations such as centrifugation speed and the resulting concentration of PRP/PRF [43]. Table-2 shows the different influences and clinical considerations of PRP and PRF based on patient-specific and procedural factors.

Age is a significant variable, as it impacts both the cellular activity and the concentration of growth factors available within PRP and PRF [44]. With advancing age, individuals experience a natural decline in the proliferative capacity of cells, including MSCs and osteoblasts, which are crucial for bone formation and repair [45]. Studies indicate that with age, the activity of MSCs diminishes due to increased cellular senescence and oxidative stress, leading to reduced proliferative and differentiation capacity [46].

Moreover, the concentration of essential growth factors, such as insulin-like growth factor (IGF-1), platelet-derived growth factor (PDGF), and vascular endothelial growth factor (VEGF), is often lower in elderly patients, further hindering the efficacy of regenerative therapies such as PRP and PRF [44]. Studies demonstrate that IGF-1 and related growth factors can partially reverse age-related declines in cell function and improve the efficacy of stem cell-based regenerative approaches, suggesting a potential benefit for pairing PRP and PRF with adjunctive growth factor therapies in older populations

Also, bone quality plays a critical role in the effectiveness of these platelet concentrates. Patients with high bone density tend to exhibit better initial stability and faster osseointegration, while those with low bone density or osteoporosis may show reduced responsiveness to PRP/PRF treatments due to compromised structural integrity and lower baseline bone regeneration capacity [43].

Lower bone density provides a less supportive environment for osseointegration, leading to reduced implant stability, increased risk of micromovement, and a delayed healing process [47]. For these patients, higher concentrations of PRF or repeated applications may be necessary to enhance growth factor availability and sustain a conducive healing environment, promoting better cellular response and bone regeneration over time [48].

In contrast, patients with high bone density typically offer a robust structural foundation that facilitates immediate implant stability and a faster integration process [49]. For these individuals, standard PRF protocols without additional modifications are often sufficient, as the natural bone quality already supports rapid healing and effective osseointegration [34].

In addition, health conditions such as diabetes, cardiovascular disease, and autoimmune disorders can significantly influence the effectiveness of PRP and PRF in promoting osseointegration [44]. These conditions often impair healing and reduce blood supply to the implant site, which can limit the regenerative benefits typically provided by both methods [50]. For example, diabetes is associated with compromised vascular function and delayed wound healing, which may slow osseointegration and reduce the bioactivity of PRP and PRF[51].

Furthermore, Implant location is another crucial factor, as areas with high vascularity and favorable bone quality (such as the anterior mandible) typically respond better to PRP and PRF applications than regions with less vascular support or reduced bone density (such as the posterior maxilla) [1]. Enhanced vascularity facilitates the delivery of nutrients and oxygen, which are essential for cell proliferation and bone remodeling, thus improving the efficacy of both methods [50]. Systemic health conditions, including diabetes, cardiovascular disease, and autoimmune disorders, can also significantly impact the success of PRP and PRF treatments. These conditions often impair healing, reduce blood supply, and affect immune function, potentially diminishing the bioactivity of both and their capacity to support osseointegration [43].

Technical aspects such as centrifugation speed and duration play a substantial role in determining the quality and efficacy of PRP and PRF[30]. Centrifugation protocols directly influence the concentration of platelets, growth factors, and leukocytes in the final product, and variations in speed and duration can yield significant differences in PRP/PRF composition [52]. Higher centrifugation speeds generally result in more platelet-poor plasma, while lower speeds may retain more platelets and growth factors but reduce the concentration of fibrin in PRF [53]. Achieving the ideal balance is essential, as excessively high or low concentrations of platelets and fibrin can impact the biological activity of both [54]. Standardized protocols for centrifugation are still lacking, which contributes to variability in outcomes across studies and clinical applications [45].

The concentration of PRP and PRF is another technical variable that affects their regenerative potential. The appropriate concentration may vary depending on patient-specific factors and the clinical context, further highlighting the need for individualized treatment protocols [55].

## Challenges and Limitations

While PRP and PRF have shown promise as effective in enhancing osseointegration in dental implantology, several challenges and limitations persist in the current body of research [56]. Many studies investigating the efficacy of both methods are limited by small sample sizes, which can reduce the statistical power needed to draw reliable conclusions and limit the generalizability of findings to broader patient populations [57][58]. Also, a lack of standardization in study design especially regarding PRP and PRF preparation protocols introduces variability that complicates the comparison of results across studies [59]. This variability is compounded by differences in centrifugation speeds, platelet concentrations, and application methods, all of which impact the biological properties of both and, subsequently, their clinical effectiveness [43].

Additionally, the lack of anticoagulants in PRF preparation can lead to clot formation during centrifugation, which may affect its handling and application consistency in clinical settings [45].

Together, these challenges underscore the need for more rigorous, well-designed studies that address the variability in PRP and PRF preparation, establish standardized protocols, and provide long-term data on their effects on dental implantology [43].

## Perspective of Clinical Implications

Advances in regenerative dentistry call for further research on PRP and PRF, particularly randomized clinical trials with large samples and standardized preparation protocols, to optimize their application in implantology [1].

These trials should include clear definitions of centrifugation speeds, platelet concentrations, and application methods to reduce variability and enhance the comparability of findings [59]. Standardizing these protocols will allow for more reliable conclusions about the efficacy of PRP and PRF, enabling researchers to determine the optimal preparation and application methods for specific clinical scenarios [3].

Moreover, future trials should prioritize long-term follow-up to assess the durability of PRP and PRF effects on osseointegration and implant stability over extended periods, as current evidence is limited primarily to short- and medium-term outcomes [60].

Another critical area for future research is the exploration of PRP and PRF efficacy across diverse patient populations with varying systemic health conditions, bone quality, and implant locations. Given that age, systemic health, and other patient-specific factors can significantly influence the regenerative potential of PRP and PRF, more research is needed to identify which subgroups may benefit most from these treatments [32]. This individualized approach would help clinicians make more informed decisions about when and for whom PRP and PRF may offer the greatest benefit [31][20].

To make the application of PRP and PRF more accessible, training programs should be developed for dental professionals, providing education on best practices for the preparation, handling, and clinical use of these materials [54]. Moreover, practitioners should remain mindful of the cost implications and weigh the benefits of PRP and PRF against the financial considerations and individual patient needs [32]. In cases where both methods may be particularly beneficial such as in elderly patients, those with poor bone quality, or those with systemic conditions affecting bone healing clinicians can use these platelet concentrates as valuable adjuncts to improve patient outcomes [61].

So, more high-quality research is conducted and standardized protocols become established, PRP and PRF have the potential to become routine elements of dental implantology. By refining application methods and targeting specific patient groups most likely to benefit, practitioners can enhance the efficacy of implant procedures, ultimately improving patient satisfaction and implant longevity.

## Conclusion

In recent years, PRP and PRF have emerged as promising biological adjuncts in dental implantology, showing the potential to enhance the critical process of osseointegration. Both PRP and PRF deliver concentrated growth factors, cytokines, and a structural matrix that can accelerate bone healing and promote a robust bone-to-implant interface. Clinical and preclinical studies indicate that these autologous platelet concentrates can improve early implant stability, reduce healing times, and enhance bone regeneration, particularly in patients with compromised bone quality or systemic conditions affecting healing.

Key findings from the current literature underscore the biological mechanisms by which PRP and PRF contribute to bone regeneration. PRP’s rich composition of growth factors, such as PDGF, TGF-β, and VEGF, initiates cellular proliferation and angiogenesis, vital for bone integration. PRF’s unique fibrin matrix provides a sustained release of these growth factors, offering a long-lasting regenerative effect while promoting soft tissue healing and reducing inflammatory responses. While PRP and PRF share similar components, their differences in preparation and release profiles may suit them to distinct clinical applications, though further comparative studies are needed to clarify these roles. Despite promising preliminary results, a significant limitation in the current literature is the lack of long-term data on the efficacy of PRP and PRF in implant osseointegration. To establish reliable protocols, future research should focus on well-structured, large-scale studies with consistent methodologies. This will facilitate robust conclusions regarding long-term outcomes and support evidence-based clinical practice. Overall, PRP and PRF offer promising support for enhancing osseointegration, particularly in patients with bone healing challenges. With ongoing validation, PRP and PRF could become integral in implant procedures, broadening success rates and improving patient outcomes across varied populations.

## Conflict of Interest

None declared.
